# Practice with Compassion: Preparing for Real-World Psychiatry through Simulated Patient Training

**DOI:** 10.1192/j.eurpsy.2026.10958

**Published:** 2026-07-31

**Authors:** J. D. Fekete, A. Hambuch, K. Eklicsné Lepenye, T. Tényi, P. Osváth, Á. Nagy, S. Fekete, V. Vörös

**Affiliations:** 1 Department of Languages for Biomedical Purposes and Communication; 2 Department of Psychiatry and Psychotherapy, University of Pécs Medical School, Pécs, Hungary

## Abstract

**Introduction:**

Simulation-based training with standardized patients (SPs) has emerged as a powerful tool in psychiatric education, providing a safe, controlled environment for learners to practice complex clinical interactions. Recent evidence confirms that this approach can reduce trainee anxiety and improve confidence, knowledge, empathy, and clinical communication skills. Such experiential learning is crucial in psychiatry, given the sensitive, interpersonal nature of psychiatric interviews and the need to build therapeutic rapport in a risk-free setting.

**Objectives:**

To evaluate the educational impact of SP-assisted psychiatric interview training for medical students and to gather insights for refining this methodology in future psychiatry curricula.

**Methods:**

60 fifth-year medical students (German and English-language programs) at the Medical School of the University of Pécs, Hungary, participated in an SP-based training module. In groups of three, they conducted a simulated psychiatric interview with an SP portraying a challenging scenario (e.g., a patient with major depressive disorder with suicidal ideations). Faculty observers were present to ensure fidelity and support. Each session included structured debriefing with SPs providing feedback from the “patient’s” perspective, focusing on emotional impact and communication. Students also completed an anonymous online questionnaire, with both objective items and open reflections. Survey participation had no effect on assessment outcomes.

**Results:**

Students initially reported notable apprehension and self-doubt, citing performance pressure, fear of inadequacy, and discomfort with being observed. Some who began the course feeling confident later recognized gaps in their clinical interviewing skills. Through repeated SP encounters and personalized feedback, learners became more self-aware and comfortable in managing psychiatric interviews. By the end of the course, participants highly valued the hands-on experience, reporting significant improvements in communication skills, empathy, and ability to manage time pressures during interviews. Feedback from the SPs also validated many of the students’ existing strengths and highlighted their progress, reinforcing the learning outcomes.

**Conclusions:**

Psychiatric interviews are complex and sensitive; SP-based simulation provides a safe, impactful method for skill development. It exposes students to diverse conditions while protecting patient safety. This approach fosters empathetic communication, active listening, and therapeutic rapport, while encouraging reflective practice through immediate feedback. Integrating SPs into psychiatry training offers a dynamic, immersive learning experience that strengthens clinical competence and preparedness for real-world psychiatry, supporting broader evidence on the value of simulation in medical education.

**Disclosure of Interest:**

None Declared

